# Supplementary material for: Identification of photoperiod-induced specific miRNAs in the adrenal glands of Sunite sheep (Ovis aries)
Source: Front Vet Sci. 2022 Jul 22;9:888207. doi: 10.3389/fvets.2022.888207 (PMC9354845; doi:10.3389/fvets.2022.888207)
Supplement: Supplementary file 1 [file Data_Sheet_1.ZIP › Supplementary file/miRDeep2 file.zip!/miRDeep2 file/Know_Structure/LP42A2/oar-mir-411a.pdf]

miRBase precursor : oar-mir-411a
Total read count : 41206
oar-miR-411a-5p read count : 39569
oar-miR-411a-3p read count : 1628
remaining reads : 9

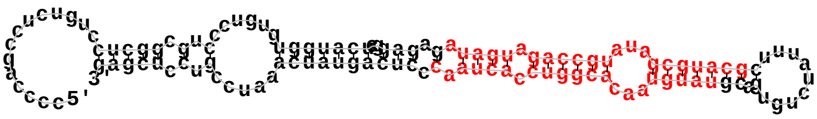

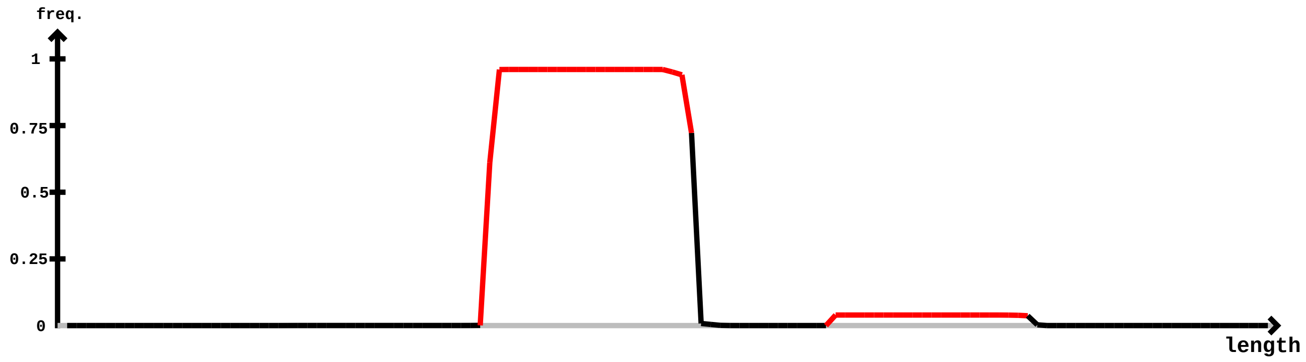

oar-miR-411a-3p

oar-miR-411a-5p

```
5'- ccccagccucuguccucggcgucccugugugguacucggagagauaguagaccguauagcguacgcuuuaucugugacguauguaacacgguccacuaacccucaguaucaaauccguccucgag   -3'    exp
    ..............(((((.(.(......(((((((..(((.(.((((.((((((...((((((((.........).)))))))...)))))).)))).).))))))))))).....).).)))))   reads   mm
    ................cggcgucccugugugguacucggagaga.................................................................................   1       0
    ......................cccugugugguacucggaga...................................................................................   1       0
    ......................cccugugugguacucggagaga.................................................................................   3       0
    ......................cccugugugguacucggagagau................................................................................   1       0
    .......................ccugugugguacucggagaga.................................................................................   1       0
    ........................cugugugguacucggagaga.................................................................................   1       0
    .........................................agauaguagaccguauagcgu...............................................................   2       0
    .........................................agauaguagaccguauagcgua..............................................................   1       0
    .........................................agauaguagaccguauagcguac.............................................................   1       0
    ..........................................gauaguagaccguauagcgu...............................................................   3       0
    ..........................................gauaguagaccguauagcgua..............................................................   1       0
    ..........................................gauaguagaccguauagcguac.............................................................   4       0
    ..........................................gauaguagaccguauagcguacg............................................................   9       0
    ...........................................auaguagaccguauagcg................................................................   2       0
    ...........................................auaguagaccguauagcgu...............................................................   332     0
    ...........................................auaguagaccguauagcgua..............................................................   301     0
    ...........................................auaguagaccguauagcguac.............................................................   5597    0
    ...........................................auaguagaccguauagcguacg............................................................   18827   0
    ...........................................auaguagaccguauagcguacgc...........................................................   68      0
    ...........................................auaguagaccguauagcguacgcu..........................................................   48      0
    ...........................................auaguagaccguauagcguacgcuu.........................................................   3       0
    ...........................................auaguagaccguauagcguacgcuuu........................................................   1       0
    ............................................uaguagaccguauagcg................................................................   1       0
    ............................................uaguagaccguauagcgu...............................................................   48      0
    ............................................uaguagaccguauagcgua..............................................................   141     0
    ............................................uaguagaccguauagcguac.............................................................   3383    0
    ............................................uaguagaccguauagcguacg............................................................   10581   0
    ............................................uaguagaccguauagcguacgc...........................................................   81      0
    ............................................uaguagaccguauagcguacgcu..........................................................   91      0
    ............................................uaguagaccguauagcguacgcuu.........................................................   27      0
    ............................................uaguagaccguauagcguacgcuuu........................................................   3       0
    .............................................aguagaccguauagcguac.............................................................   2       0
    .............................................aguagaccguauagcguacg............................................................   8       0
    .............................................aguagaccguauagcguacgc...........................................................   1       0
```

```
                                                                               oar-miR-411a-3p
                                           oar-miR-411a-5p
ccccagccucuguccucggcgucccugugugguacucggagagauaguagaccguauagcguacgcuuuaucugugacguauguaacacgguccacuaacccucaguaucaaauccguccucgag

.............................................aguagaccguauagcguacgcu..........................................................   2      0
.................................................................cuuuaucugugacguauguaacacgguccacuaac.........................   1      0
.............................................................................cguauguaacacgguccacuaa..........................   1      0
...............................................................................uauguaacacgguccacu............................   13     0
...............................................................................uauguaacacgguccacua...........................   30     0
...............................................................................uauguaacacgguccacuaa..........................   73     0
...............................................................................uauguaacacgguccacuaac.........................   1396   0
...............................................................................uauguaacacgguccacuaacc........................   113    0
...............................................................................uauguaacacgguccacuaaccc......................   1      0
................................................................................auguaacacgguccacuaac.........................   1      0
```
